# Adaptive dose painting for prostate cancer

**DOI:** 10.3389/fonc.2022.973067

**Published:** 2022-09-27

**Authors:** Emil Fredén, David Tilly, Anders Ahnesjö

**Affiliations:** ^1^ Department of Oncology, Södersjukhuset, Stockholm, Sweden; ^2^ Department of Genetics, Immunology and Pathology, Medical Radiation Sciences, Uppsala University, Uppsala, Sweden; ^3^ Department of Medical Physics, Uppsala University Hospital, Uppsala, Sweden

**Keywords:** dose painting, dose-response modeling, adaptive radiation therapy, prostate cancer, MR-linac

## Abstract

**Purpose:**

Dose painting (DP) is a radiation therapy (RT) strategy for patients with heterogeneous tumors delivering higher dose to radiation resistant regions and less to sensitive ones, thus aiming to maximize tumor control with limited side effects. The success of DP treatments is influenced by the spatial accuracy in dose delivery. Adaptive RT (ART) workflows can reduce the overall geometric dose delivery uncertainty. The purpose of this study is to dosimetrically compare ART and non-adaptive conventional RT workflows for delivery of DP prescriptions in the treatment of prostate cancer (PCa).

**Materials and methods:**

We performed a planning and treatment simulation study of four study arms. Adaptive and conventional workflows were tested in combination with DP and Homogeneous dose. We used image data from 5 PCa patients that had been treated on the Elekta Unity MR linac; the patients had been imaged in treatment position before each treatment fraction (7 in total). The local radiation sensitivity from apparent diffusion coefficient maps of 15 high-risk PCa patients was modelled in a previous study. these maps were used as input for optimization of DP plans aiming for maximization of tumor control probability (TCP) under rectum dose constraints. A range of prostate doses were planned for the homogeneous arms. Adaptive plans were replanned based on the anatomy-of-the-day, whereas conventional plans were planned using a pre-treatment image and subsequently recalculated on the anatomy-of-the-day. The dose from 7 fractions was accumulated using dose mapping. The endpoints studied were the TCP and dose-volume histogram metrics for organs at risk.

**Results:**

Accumulated DP doses (adaptive and conventional) resulted in high TCP, between 96-99%. The largest difference between adaptive and conventional DP was 2.6 percentage points (in favor of adaptive DP). An analysis of the dose per fraction revealed substantial target misses for one patient in the conventional workflow that—if systematic—could jeopardize the TCP. Compared to homogeneous prescriptions with equal mean prostate dose, DP resulted in slightly higher TCP.

**Conclusion:**

Compared to homogeneous dose, DP maintains or marginally increases the TCP. Adaptive DP workflows could avoid target misses compared to conventional workflows.

## 1 Introduction

When a tumor’s radiation sensitivity is heterogeneous, radiation therapy (RT) with conventional homogeneous dose prescriptions will not maximize the tumor’s response per delivered radiant energy (“integral dose”). Under the assumption that individual cancer cells respond independently to each other, several authors have shown theoretically that stronger curative effects per delivered dose is achieved by prescribing higher dose to radiation resistant tumor sub-volumes and less to sensitive ones (1, 2). With an overall reduction of dose, it is assumed that the overall risk for side effects can be reduced. The approach of differentiating the tumor dose over its sub-volumes requires that pretreatment functional imaging (3) can be used to spatially map a quantity that correlates with radiation sensitivity. The spatial differentiation of dose prescriptions on a per voxel basis has been referred to as ‘dose painting by numbers’ (DPBN) (4). By building upon the work of Vogelius et al. (5), Grönlund et al. (6) developed a *failure-driven* DPBN formalism incorporating clinical endpoint data and information from functional imaging. Their formalism was applied to prostate cancer (PCa) in a simulation study based on imaging of the apparent diffusion coefficient (ADC) with MRI (7) for which they used a correlation between ADC and an assumed Gleason score (GS) (8) as an intermediate step for scoring and modelling dose-response variations. The tumor dose-response was then modelled based on treatment failure frequencies versus biopsied GS from a retrospective study of patients treated with homogeneous dose (9, 10). The feasibility of delivering spatially differentiated DPBN plans for PCa patients was investigated in a follow up simulation study considering dose delivery uncertainties (11). They also concluded that the potential of dose painting increases as the geometric uncertainties of treatment delivery decrease.

The MR-linac (MRL) enables an adaptive workflow taking advantage of the soft-tissue contrast of magnetic resonance imaging (12). A key feature of the MRL is the ability to perform plan adaptation prior to each given fraction based on imaging of the patient in treatment position. The present work aims to investigate if the reduced geometric uncertainties obtained by adaptive RT (ART) can increase the potential of dose painting compared to conventional, non-adaptive, treatment workflows. To this end, we present a treatment simulation study where the DPBN formalism for PCa by Grönlund et al. (7, 11) is combined with adaptive workflow features provided by the Elekta Unity MRL system (13). For reference we also included study arms with homogeneous dose escalation. The more peaked dose distributions used in prostate SBRT (14) could be interesting as reference as well. However, to avoid bias caused by the arbitrariness of SBRT dose max locations we preferred homogeneous dose arms as reference. As primary endpoint we used the calculated tumor control probability (TCP), and as secondary endpoints dose-volume histogram (DVH) based metrics for the dose to organs at risk (OARs). Previous studies have investigated adaptive dose painting strategies for head and neck cancers (15), but to our knowledge the present simulation study is the first to combine DPBN with daily replanning for PCa.

## 2 Materials and methods

### 2.1 Overview of study design

In the present treatment simulation study, we investigate if an adaptive RT workflow can increase the potential of dose painting in terms of TCP and/or reduced dose to risk organs. For planning and treatment simulation we used a research version of a commercial treatment planning system (TPS) (RayStation 10.1.130.16, RaySearch Laboratories, Stockholm, Sweden) together with purpose designed scripts. We simulated two different dose prescription strategies (homogeneous dose vs. DPBN) combined with two different treatment delivery workflows (conventional vs. adaptive), i.e., in total four study arms labelled: Homo-conv for homogeneous dose with conventional delivery, Homo-adap for homogenous dose with adaptive delivery, DPBN-conv for dose painting by numbers with conventional delivery, and DPBN-adap for dose painting by numbers with adaptive delivery. The DPBN-conv and DPBN-adap plans were *constrained by rectum dose-volume criteria*, but no upper limit was set on the dose to individual voxels of the prostate, i.e. these plans had the planning aim *‘treat-to-tolerance’*. The homogeneous dose plans were optimized towards fixed target dose, aiming at high target coverage while keeping the dose to the rectum as low as possible. For the homogeneous arms we thus implemented target coverage constraints together with rectum objectives. We optimized a set of homogeneous plans with a range of target dose levels to study the relationship between target dose, rectum load, and tumor control, and to set the DPBN plans in a clinical context. Our study design differs from that of Grönlund et al. (11) who kept the mean dose to the prostate equal between the homogeneous and dose painted plans; moreover, they only simulated conventional treatment flows. The resulting TCP and rectum DVH metrics were calculated based on the accumulated dose from simulated full treatment courses; these metrics were used to compare the four study arms. **Table 1** summarizes the key parameters for the study arms. A more detailed description is given in the following sctions.

**Table 1:** The four study arms simulated.

	Homo-conv homogeneous doseconventional delivery	Homo-adap homogeneous doseadaptive delivery
Plan generation	Reference plan	Plan of the day
Margin	6 mm	3 mm
Target *constraints*	*D*_98%_ > 0.95*D*_p_, *D*_2%_< 1.05*D*_p,_ D_p_ є{43.89,44.89,…,60.89}Gy	
Rectum *objectives*	*V*_33Gy_<30%, *V*_38Gy_<15%, *V*_41Gy_<10%	
	
	**DPBN-conv** dose paintingconventional delivery	**DPBN-adap **dose paintingadaptive delivery
Plan generation	Reference plan	Plan of the day
Minimax optimization	6 mm	3 mm
Target goal	Maximize TCP	
Rectum *constraints*	*V*_33Gy_<30%, *V*_38Gy_<15%, *V*_41Gy_<10%	

For the two arms with homogeneous dose, the plans were designed based on rectum dose objectives that can be violated with a penalty in favor of covering the target with the homogeneous prescription dose *D*_p_, while for the dose painting by numbers plans, we used rectum dose constraints that cannot be violated.

### 2.2 Patient data and case generation

**Figure 1:**
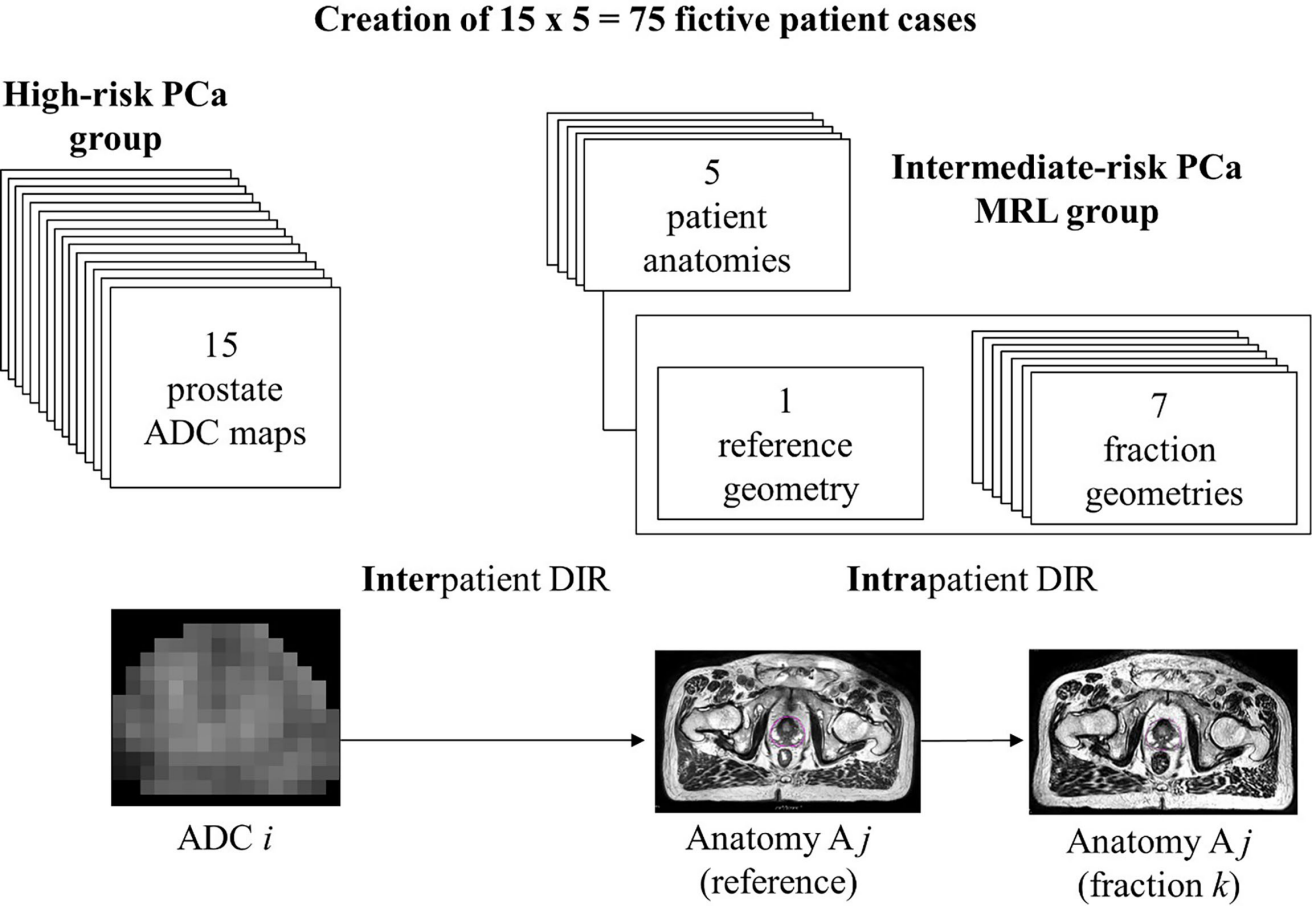
Data from two different patient groups were combined to generate images to represent 75 test cases. For the 5 patients from the group consisting of intermediate-risk PCa patients treated on Elekta Unity MRL (i.e., ‘MRL group’), we had 1 reference geometry and 7 fraction geometries. From the second group, we had ADC maps of 15 high-risk PCa patients. The bottom part of the figure shows the process of deforming the ADC maps for patients of the high-risk PCa group to the reference geometry for a patient from the MRL group. The (deformed) reference ADC maps were subsequently deformed to fit the 7 fraction geometries. Patients in the MRL group are labelled A1-A5.

For this project we had access to two sets of patient data from which we constructed 75 fictive PCa cases by fusing image data sets from the two groups. The first set consisted of images for 5 intermediate-risk PCa patients that had been treated to 42.7 Gy with hypofractionation (6.1 Gy×7 fx) on the Elekta Unity MRL at Akademiska sjukhuset ethical approval reference number: 2019–03050 (Uppsala, Sweden). For each of these 5 patients (A1-A5) we had access to one reference T2w MRI (acquired prior to treatment) and 7 fractions of T2w MRI, as well as the corresponding structure sets including the prostate, seminal vesicles (SV), rectum, bladder, anal canal, penile bulb, and femoral heads. The intrapatient anatomical variations captured in these 8 image sets allowed us to simulate a full hypofractionated treatment course of 7 fractions. We selected 15 patients included in the PARAPLY phase 2 trial with Umeå board ethical approval reference numbers 2013/154-31 and 2015/ 75-32 from the high-risk PCa patient group included in Grönlund’s previous works (7, 11) and used the ADC maps of their prostates. These 15 ADC maps were registered and fused to each of the 5 patient reference geometries from the MRL patient group through deformable image registration (DIR), resulting in planning reference ADC maps for 15×5 = 75 fictive PCa cases. In this work, a *case* is defined as the *combination of a specific patient anatomy and a single realistic prostate ADC (spatial) distribution for a high-risk PCa*. The thus fitted ADC maps were then through a subsequent intrapatient DIR operation assigned to each fraction’s geometry, thus assuming that the pre-treatment ADC values were invariantly determining the Gleason values over the full treatment course. All ADC distributions were visually checked after the transformations to minimize the risk for artifacts entering into the image data flow. In addition, the mean and spread of the ADC distributions were evaluated both before and after a deformation for validation. A schematic overview of the process is shown in **Figure 1**.

### 2.3 Treatment simulation of a case

**Figure 2:**
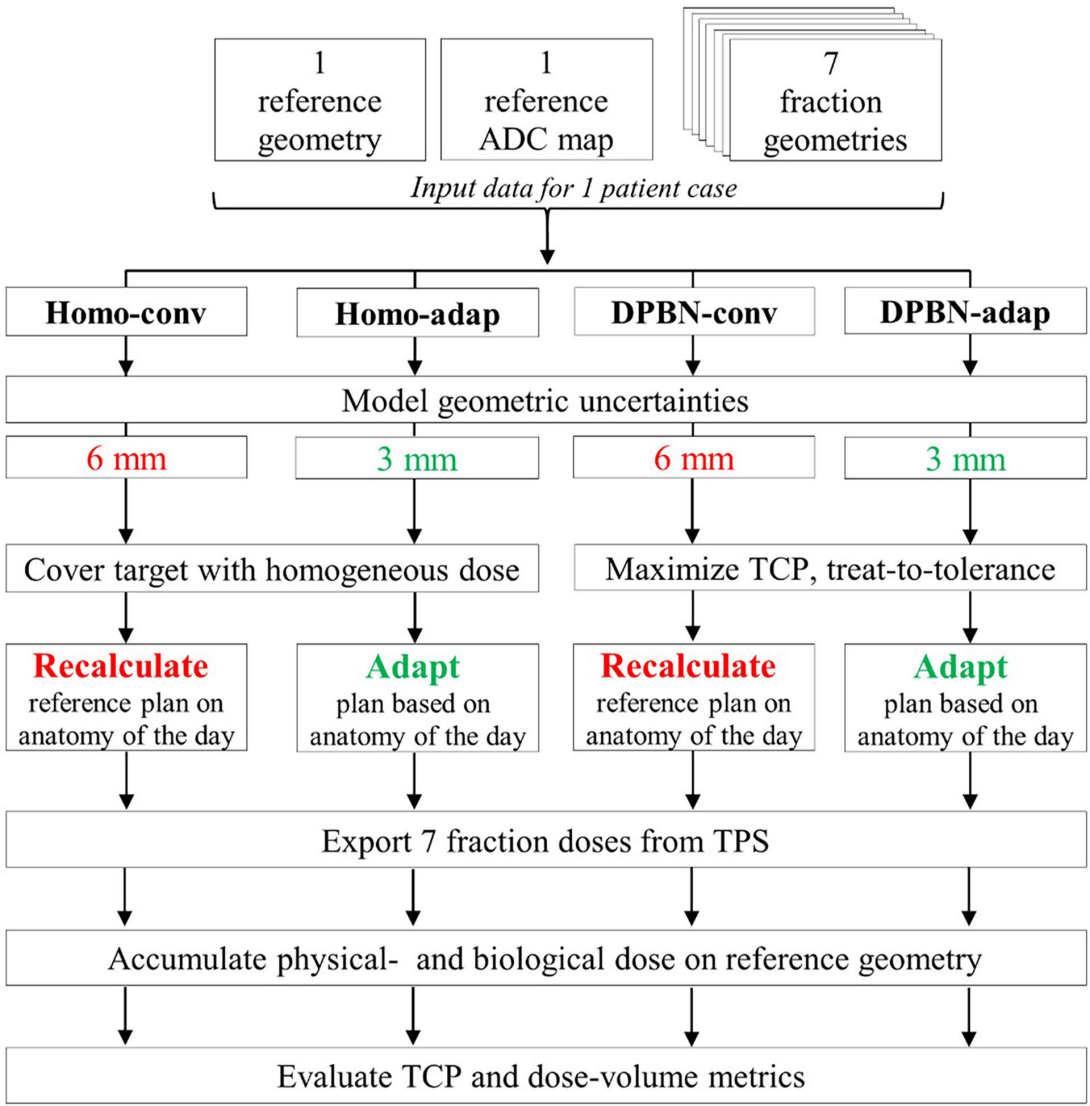
Process chart for treatment simulation of a patient case showing the key differences between the four simulated study arms. The CTV to PTV margins for the two treatment workflows were 3 mm vs 6 mm, respectively. Conventional plans (Homo-conv, DPBN-conv) were optimized using the reference geometry and were subsequently recalculated on the anatomy-of-the-day. Adaptive plans (Homo-adap, DPBN-adap) were optimized using the anatomy-of-the-day. The 7 fraction doses were exported and finally accumulated on the reference geometry to facilitate evaluation of primary TCP- and secondary DVH endpoints.

An overview of the simulation flow for the four study arms is shown in **Figure 2**. The main operations include generation of a treatment plan, modelling of the geometric uncertainties, and finally dose accumulation over all treatment fractions for endpoint calculation of the TCP and DVH metrics.

#### 2.3.1 Setup of treatment plans

Optimized treatment plans were created for all four study arms and for each of the 75 cases. All were planned for hypofractionation (7 fractions), with 7-field IMRT (static MLC, 70 segments in total). The gantry angles were set equal to those clinically used for the MRL treatments. The Homo-adap and DPBN-adap plans were optimized on the anatomy-of-the-day, whereas the conventional Homo-conv and DPBN-conv plans were planned on each patient’s reference geometry. The conventional plans were subsequently recalculated on each fraction image based on rigid CTV-to-CTV translations to simulate treatments with the field setup translated based on gold marker registrations. For the conventional plans, the isocenter was set at the volumetric center of the prostate CTV. For the adaptive plans, we extracted the isocenters from the clinical MRL plans as the fixed isocenter cannot in general be placed in the target volume. Dose calculations were performed with Monte Carlo (1% uncertainty, 3 mm voxel size). Appropriate tissue compositions and densities were assigned to the body and bony anatomy to facilitate the calculation of dose. As the patient couch of Elekta Unity is highly attenuating, the bed was included in the dose calculations for the MRL workflow, and the angle intervals with the highest change in attenuation were avoided as per clinical practice. Magnetic field effects modelled as described by Malkov and Rogers (16) had been added to the Monte Carlo engine of the TPS (17).

#### 2.3.2 Modelling of the geometric uncertainties

A major advantage of adaptive workflows is the ability to compensate at plan generation for interfraction organ deformations. The increased accuracy for adaptive workflow patients has two benefits: 1) the margins added to form the planning target volume (PTV) for generation of homogeneous plans can be reduced to lower the risk of inducing normal tissue toxicities, and 2) it has been shown that larger TCP increases can be obtained through dose painting when geometric dose delivery uncertainties are small (11).

We divided the overall geometric uncertainty of the two treatment workflows into subcomponents and assigned to each an estimate of the standard deviation (SD) based on published data. For both workflows, these subcomponents included the residual positioning errors resulting from intrafraction motion (SD 1 mm) (18), interobserver target delineation variations (SD 1 mm) (19), and an estimate of the finite precision of the treatment machine (1 mm). The residual effect of interfraction prostate deformation was only considered for the conventional workflow for which it was assigned an SD of 1 mm (20); in the adaptive workflow, prostate deformation was taken into account *via* redelineation of the prostate on the anatomy-of-the-day. For the conventional workflows, we also added the combined effect of image registration- and table translation uncertainties, which was estimated to have an SD of 1 mm (19). The SD of different components were added in quadrature and used as input to margin calculations based on van Herk’s margin formula (21) in which systematic uncertainties (preparation errors) are denoted by Σ and random uncertainties (daily patient setup variations) are denoted by sigma Since we planned for hypofractionation (7 fractions), we used van Herk’s formula


(1) $$M\left(\;\Sigma_{\text{eff}},{\text{σ}}_{\text{eff}}\right)\;=2.5\;{\text{Σ}}_{\text{eff}}+0.7{\text{σ}}_{\text{eff}}$$

with *effective* uncertainty components (22, 23), adjusted to consider the finite number of treatment fractions *N*:


(2) $$\Sigma_{eff}^{2}=\Sigma^{2}+\frac{\sigma^{2}}{N},\;\;\;\;\;\;\;\;\sigma_{eff}^{2}=\;\sigma^{2}\left(1-\frac{1}{N}\right).\;$$

The resulting (isotropic) prostate margins calculated with equations (1, 2) for the adaptive and conventional workflows were 3 mm and 6 mm, respectively. For the SV, we used the lower (LR: 5, AP: 7, SI: 7 mm) and upper limit (LR: 6, AP: 9, SI: 9 mm) of the anisotropic margins specified in the ESTRO reference (20) for the adaptive and conventional workflows, respectively. Homogeneous plans were optimized with standard CTV-to-PTV margins whereas DPBN plans were created using minimax optimization (24) with “robustness distances” set to the same values as the CTV-to-PTV margins; the minimax optimizer generates a set of treatment scenarios with patient setup displacements along three axes, and aims to find a plan which is optimal for the worst case of these scenarios (i.e., a plan which is robust to geometric uncertainties). For each objective, the software allows it to be set as ‘robust’ or not, i.e. evaluated for all the scenarios. We selected only the TCP objective as robust.

#### 2.3.3 Homogeneous dose prescriptions

We were interested to see whether DPBN, limited by normal tissue constraints, would be superior to homogeneous dose escalation. Dose escalation to the prostate is in general limited by gastrointestinal (GI) toxicities (e.g. diarrhea, rectal bleeding, proctitis), genitourinary (GU) toxicities (e.g. dysuria, hematuria, obstruction) and erectile dysfunction (25). A set of homogeneous plans were generated with prostate prescription doses ranging from 43.89 Gy up to 60.89 Gy in increments of 1 Gy, where 43.89 Gy (EQD2 = 91.6 Gy_1.93_) corresponds to the dose level used in the previous works of Grönlund et al. (7, 11). In total, 7x18 homogeneous adaptive plans and 18 homogeneous conventional plans were optimized for each of the 5 patient anatomies. The 7x18+18 homogeneous plans were assigned to each of the 15 cases corresponding to the particular patient anatomy. The homogeneous plans per patient anatomy could be reused because no ADC information was used for planning of the homogeneous dose arms, Homo-conv and Homo-adap. For target dose uniformity, we used dose-volume *constraints* requiring that 98% of the target volume receives at least 95% of the prescribed dose, and that at most 2% of the volume receives doses larger than 105% of the prescribed dose.

#### 2.3.4 Dose painting prescriptions

For the DPBN plans, the Grönlund et al. (11) TCP formalism (summarized briefly in Appendix A) was used to maximize the TCP subject to rectum constraints. The ADC maps were downsampled to the resolution of the dose grid, and subsequently transformed to Gleason score probabilities through a ‘low precision’ ADC-to-Gleason mapping constructed by Grönlund et al. (7, 11).

#### 2.3.5 Dose to risk organs

Dose to the OARs other than the rectum could be held below the clinically set tolerance levels (femoral heads: *D*_2%_<30 Gy, bladder: *D*_mean_<34 Gy) using a single ‘dose falloff’ objective (effectively aiming for a tight dose gradient around the target volume). For the rectum, we implemented the three volume-at-dose (VaD) metrics (*V_33Gy_* < 30%, *V_38Gy_* < 15%, *V_41Gy_* < 10%, where *V_D_* is the volume of the organ receiving doses larger than *D*) as *objectives* during homogeneous plan generation, and as *constraints* during DPBN plan generation. Rectum *objectives* were used in the homogeneous arms since rectum constraints could potentially conflict with the imposed target coverage constraints. We used these rectum DVH metrics since they are clinically implemented for plan evaluation at our clinic, Akademiska sjukhuset (Uppsala, Sweden). For all study arms, we *complemented* the clinically used evaluation criteria with *D*_2%_<42.7 Gy (soft) objectives to limit high doses in the rectum, bladder and remaining normal tissues whenever possible (target goals were prioritized).

#### 2.3.6 Evaluation of TCP and DVH endpoints based on accumulated dose from a full treatment course

For each case and study arm, we evaluated the TCP and rectum DVH endpoints based on the accumulated dose from 7 fractions. Biological dose (EQD2) was accumulated to calculate TCP according to Grönlund’s formalism described in Appendix A. To be consistent with Grönlund’s earlier work we used an α/β ratio of 1.93 Gy to calculate EQD2 (7). As input to the TCP calculations, we used the accumulated EQD2, the down sampled reference ADC map, and assigned to the full vesicle volume a Gleason score of 6, which is the lowest risk category in the TCP model (this assumption was made since we did not have any ADC information for the SV). The vesicle volume was included in the TCP formalism to be able to evaluate the effect of potential SV target loss (for all study arms, the SVs were prescribed a near-min dose of 43.89 Gy corresponding to an SV control probability larger than 99%). Physical dose was accumulated to generate cumulative DVHs. Out of the OARs, we decided to mainly focus on the rectum since it is the most dose limiting organ for PCa.

#### 2.3.7 Deformable image registrations for mapping fraction doses to a common reference frame

For each case the dose was accumulated through mapping of all fraction doses to the patient’s reference frame *via* the geometric transformation determined by a DIR. The result of the DIR consists of a rigid transformation matrix describing rotations and translations and a displacement vector field (DVF) describing the deformations. The ‘hybrid DIR’ option in the TPS was used to calculate DIRs between the reference- and each of the 7 fraction geometries. ‘Hybrid DIR’ is based on the ANACONDA algorithm (26) and employs three non-linear terms: an image similarity term, a grid regularization term, and a term considering the similarity between structures delineated in the two geometries. The prostate CTV, SV, rectum, and bladder were selected as ‘controlling ROIs’ (RayStation term for registration guiding structures) with weight 0.8.

The accumulated dose accuracy is sensitive to uncertainties in the DIR (27) and therefore it is important to assess the registration quality. To this end, we calculated the DICE score (DSC) (28) and Hausdorff distance (29) for the prostate CTV, SV, rectum, and bladder. Both measures quantify the ‘similarity’ (i.e., agreement) between structures defined in two different reference frames. A dosimetric evaluation was also performed to assess the quality of the registrations for the purpose of dose accumulation. This was done by comparing (per fraction) rectum VaD evaluated before and after dose mapping.

## 3 Results

**Figure 3:**
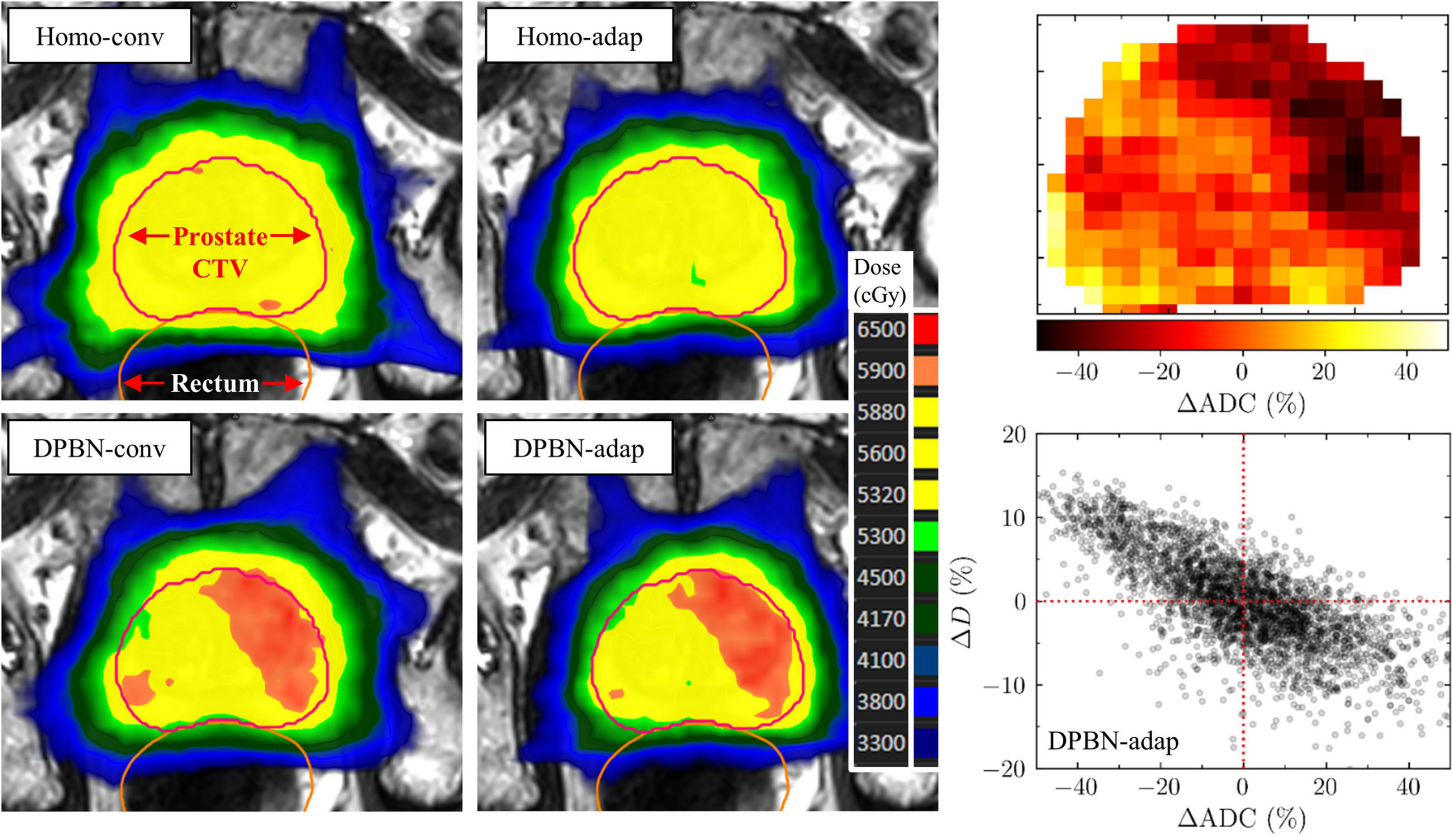
In the top 2x2 panels, transverse 2D-slices of 4 *reference* (i.e., not dose accumulated) plans are presented—showcasing the 4 respective study arms—for 1 of the 75 patient cases. The lower left panel shows the corresponding slice of the ADC map used to generate the DPBN plans. The lower right panel shows the prostate voxel dose (percentage deviation of 56 Gy) as a function of ADC (percentage deviation of ADC_mean_=1046 10^-6^mm^2^s^-1^) for the DPBN-adap reference plan.

**Figure 4:**
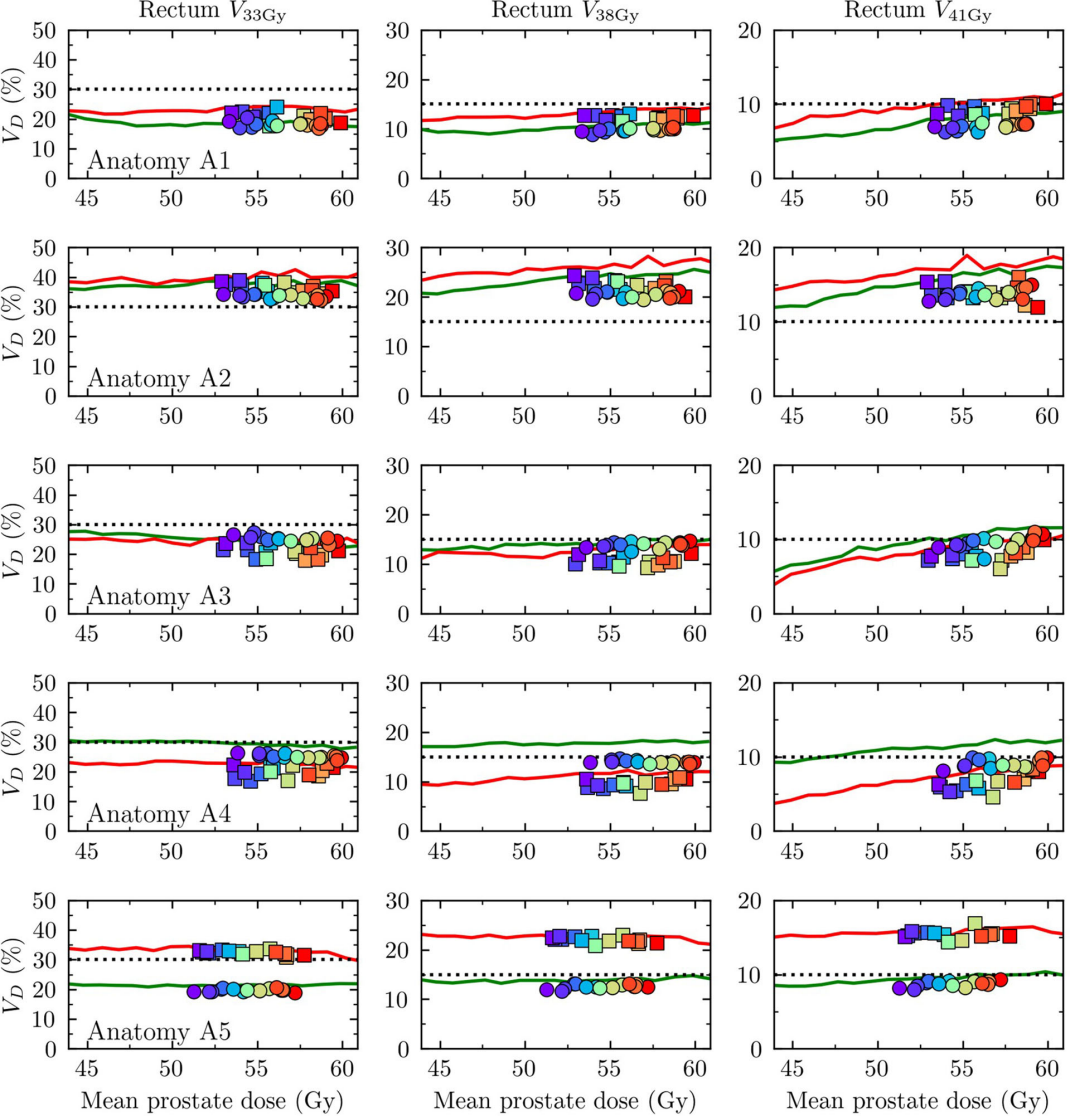
Rectum VaD as a function mean prostate dose. Each row corresponds to one of the five patient anatomies A1-A5 with A1 uppermost. The three columns correspond to *V*_33Gy_, *V*_38Gy_, *V*_41Gy_, respectively. Rectum constraints are indicated by dotted lines (*V*_33Gy_<30%, *V*_38Gy_<15%, *V*_41Gy_<10%). Homo-conv: red solid line, Homo-adap: green solid line, DPBN-conv: squares, DPBN-adap: circles. The squares/circles correspond to the 15 cases per anatomy and are color coded according to their mean Gleason score, as estimated through an ADC-to-Gleason-score probability mapping (the colormap scale is shown in **Figure 5**).

In the present work we sought to investigate the potential benefit of plan adaptation for prescriptions based on DPBN. The study arms based on homogeneous prescriptions were used for reference to set the dose painting results in a clinical context. In **Figures 3**, (4) reference plans—showcasing the different study arms—for 1 of the 75 patient cases are presented together with the ADC map used to generate the particular DPBN plans. Compared to the homogeneous dose plans, the DPBN plans have distinct high dose regions that follow a low ADC structure. According to the model, low ADC structures are indicative of radiation resistant foci.

We begin the presentation of the results with a section comparing the overall difference in TCP- and rectum DVH endpoints calculated for the *conventional- and adaptive dose painting arms*, DPBN-conv and DPBN-adap. In the following section, these results are then grouped according to patient anatomy and compared against the results from homogeneous dose escalation in Homo-conv and Homo-adap. The results presented in the first two sections were calculated based on the accumulated dose from a full treatment course, and it is evident that rectum constraints were violated despite our intention to ‘treat-to-tolerance’. Therefore, we break down the endpoint calculation per fraction in the third section to eliminate the role of dose mapping uncertainties that might confound potential differences between the conventional and adaptive workflows. In the fourth section, we then illustrate two mechanisms for the apparent rectum constraint violations. One of these mechanisms is inherent to the conventional arms, for which the use of non-representative rectum volumes can explain the observed constraint violations; the second mechanism deals with the complex task of accumulating dose to non-rigid organs that experience substantial volume changes over the treatment course. In the last section, we present the similarity measures calculated to analyze the quality of the deformable image registrations.

### 3.1 Conventional versus adaptive dose painting

Using the accumulated dose from 7 fractions, the conventionally and adaptively dose painted arms resulted in high tumor control probability for all cases (TCP: 96-99%). The difference per case between the two workflows was small; the mean difference was 0.5 percentage points, and the maximum difference was 2.6 percentage points. The adaptively dose painted arm resulted, on average, in lower rectal doses. However, analyzed per case, both negative and positive differences in rectum DVH metrics were observed (e.g., the difference in *V*_41Gy_ ranged between -7.9 and 4.5 percentage points; a negative difference implies that the adaptive workflow resulted in lower rectum dose). **Table 2** summarizes the condensed DPBN results.

**Table 2:** Comparison of the calculated endpoints for DPBN-conv and DPBN-adap.

	DPBN-conv	DPBN-adap	Δ : adap-conv
TCP (%)	98.6 [96.4, 99.3]	99.1 [98.7, 99.4]	0.5 [-0.3, 2.6]
Rectum *V*_33Gy_ (%)	26 [17, 39]	24 [17, 34]	-1.7 [-13.8, 9.4]
Rectum *V*_38Gy_ (%)	15 [8, 24]	14 [9, 21]	-1.2 [-11.1, 6.1]
Rectum *V*_41Gy_ (%)	11 [5, 17]	10 [6, 15]	-0.9 [-7.9, 4.5]

The last column (Δ) shows the difference in endpoints evaluated per case. Data are presented as mean [min, max].

### 3.2 Homogeneous dose escalation versus dose painting to tolerance

Rectal doses varied slowly as a function of mean prostate dose in the homogeneously dose escalated arms. In other words, the mean prostate dose could be escalated without substantially increasing the rectum load (in terms of the clinically used evaluation criteria). **Figure 4** shows the rectal doses as a function of mean prostate dose for all four study arms, presented separately for the five patient anatomies A1-A5. The DPBN arms resulted in equal or lower rectal doses for a given mean prostate dose compared to the homogeneous arms. For anatomies A3-A5, the DPBN-adap arm was successful in ‘treating-to-tolerance’, since at least one of the three rectum VaD metrics lies precisely on the tolerance limit and the other VaD metrics lie *on* or *below* the set tolerance limits. For anatomy A1, the rectum VaD metrics for the 15 DPBN-adap cases lie well below all three tolerance limits, and we thus failed to ‘treat-to-tolerance’. On the other end, all rectum constraints were violated for the 15 DPBN-adap cases belonging to anatomy A2. Note, however, that these results were based on the accumulated dose from 7 fractions; we further explore these results in the following sections. A further interesting observation from **Figure 4** is that the conventionally planned arms resulted in lower rectal doses compared to the adaptively planned arms for cases belonging to A3 and A4. To summarize, the outcome with regards to rectal doses from adaptive and conventional workflows depends to a large extent on the particular patient anatomy; moreover, dose painting is at least non-inferior to homogeneous dose and has the potential to decrease the rectum load. We did not prioritize near-maximum doses (*D*_2%_) for OARs in the optimization since we adopted the clinically used evaluation OAR criteria (30); however, condensed *D*_2%_ results for the four study arms are presented in **Table 3**.

**Table 3:** Comparison of near-maximum doses (*D*_2%_) for the four study arms.

	Homo-conv	DPBN-conv	DPBN-adap	Homo-adap
TCP (%)	97.9 [95.3, 99.5]	98.6 [96.4, 99.3]	99.1 [98.7, 99.4]	98.3 [95.3, 99.5]
Prostate *D*_mean_ (Gy)	55 [54, 56]	56 [52, 60]	56 [51, 60]	56 [55, 57]
Prostate *D*_2%_ (Gy)	58 [57, 59]	61 [56, 65]	61 [56, 64]	58 [56, 59]
Rectum *D*_2%_ (Gy)	50 [47, 53]	49 [45, 55]	50 [46, 55]	51 [49, 54]
Bladder *D*_2%_ (Gy)	50 [47, 55]	46 [41, 59]	45 [41, 52]	50 [47, 53]

In this comparison, a single dose level per anatomy was selected for the homogeneous arms, corresponding to the mean DPBN dose. Data is presented as mean [min, max].

**Figure 5:**
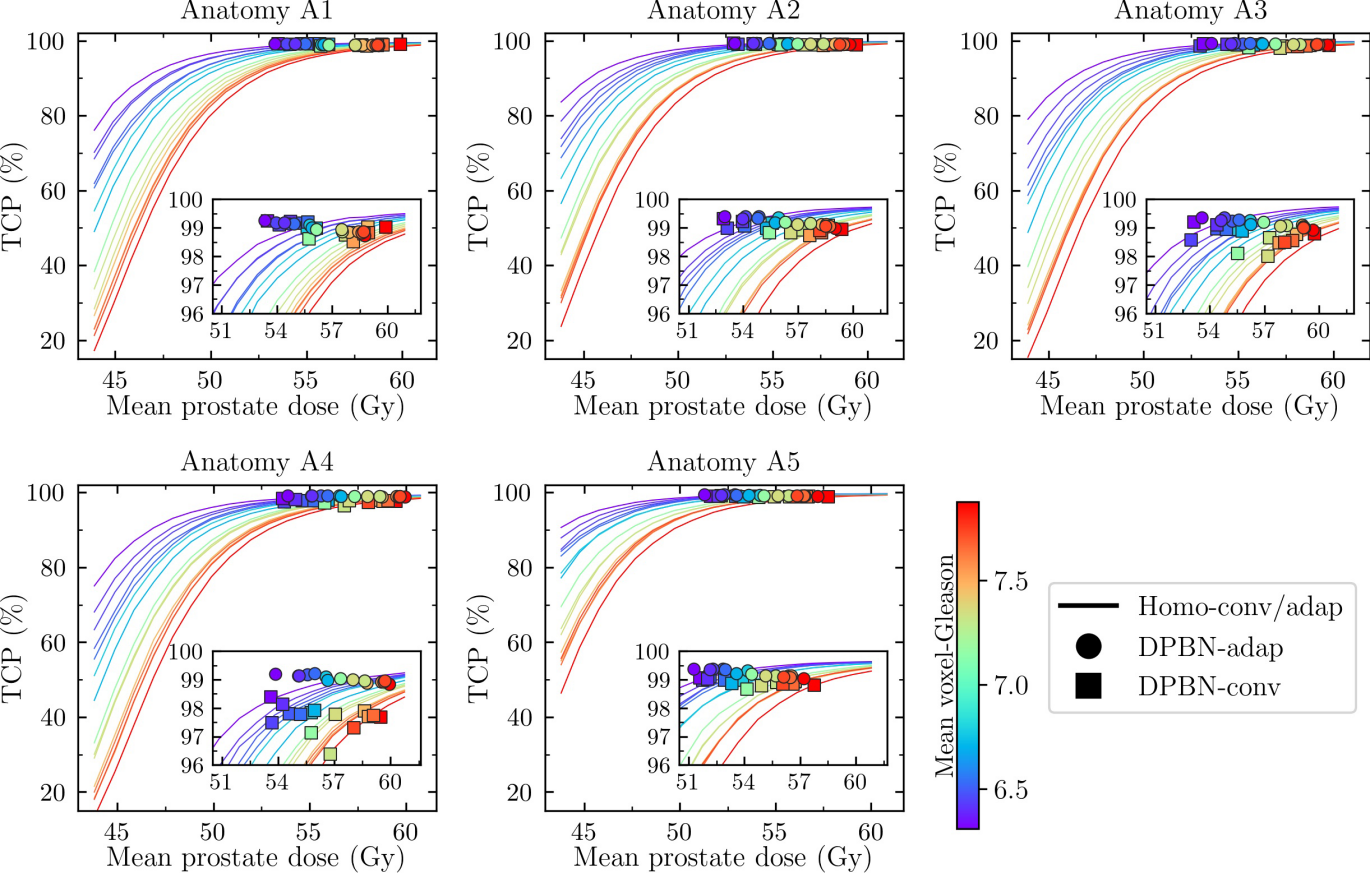
TCP as a function of mean prostate dose. Each panel corresponds to one of five patient anatomies (A1-A5). For each anatomy there are 15 cases corresponding to different prostate ADC maps. The cases are color coded according to their mean Gleason score, as estimated through an ADC-to-Gleason-score probability mapping. For a given mean prostate dose, DPBN is superior to homogeneous dose if the associated marker lies above the solid line (e.g., for cases belonging to A4, DPBN-conv markers are observed *below* the corresponding solid lines).

The dose-response curves (i.e., the TCP as a function of mean prostate dose) for the homogeneously dose escalated arms are presented separately for the five patient anatomies A1-A5 in **Figure 5** together with the resulting TCP from conventional and adaptive dose painting. Note that 15 dose-response curves were calculated for each anatomy using the same homogeneous dose but with different ADC maps; the resulting TCP is a function of dose, ADC, and implicitly a function of tumor volume (since the calculation is a product over the prostate voxels). The volume dependence explains why the dose-response curves are different for the five patient anatomies even though the same set of 15 ADC distributions were used. In the TCP model used, low ADC values indicate a high probability for high Gleason scores, and thus a worse prognosis. As is evident from **Figure 5**, the DPBN arms resulted in mean prostate doses located in the flat region of the dose-response curves. In this region there are diminishing marginal returns for additional increases in dose (i.e., the dose-response gradient is small); this explains why there is a spread in DPBN mean prostate doses resulting in similar TCP (approximately 99%) for all cases; the TPS optimizer pushed high Gleason score cases towards higher doses, since the marginal return is greater for these cases.

In **Figure 5**, the cases are color coded according to their mean Gleason score, as determined through an ADC-to-Gleason score mapping. The homogeneously dose escalated arms can be compared against the DPBN arms; DPBN is superior if the TCP for the corresponding case lies above the (homogeneous) dose-response curve at equal mean dose. For most cases, DPBN-adap and DPBN-conv was superior to homogeneous dose, except for some cases belonging to anatomy A4 for which DPBN-conv resulted in lower TCP. DPBN-adap resulted in better or similar TCP compared to DPBN-conv, with the largest difference observed for cases belonging to anatomy A4.

### 3.3 Breakdown of endpoint calculations per treatment fraction for the DPBN arms

**Figure 6:**
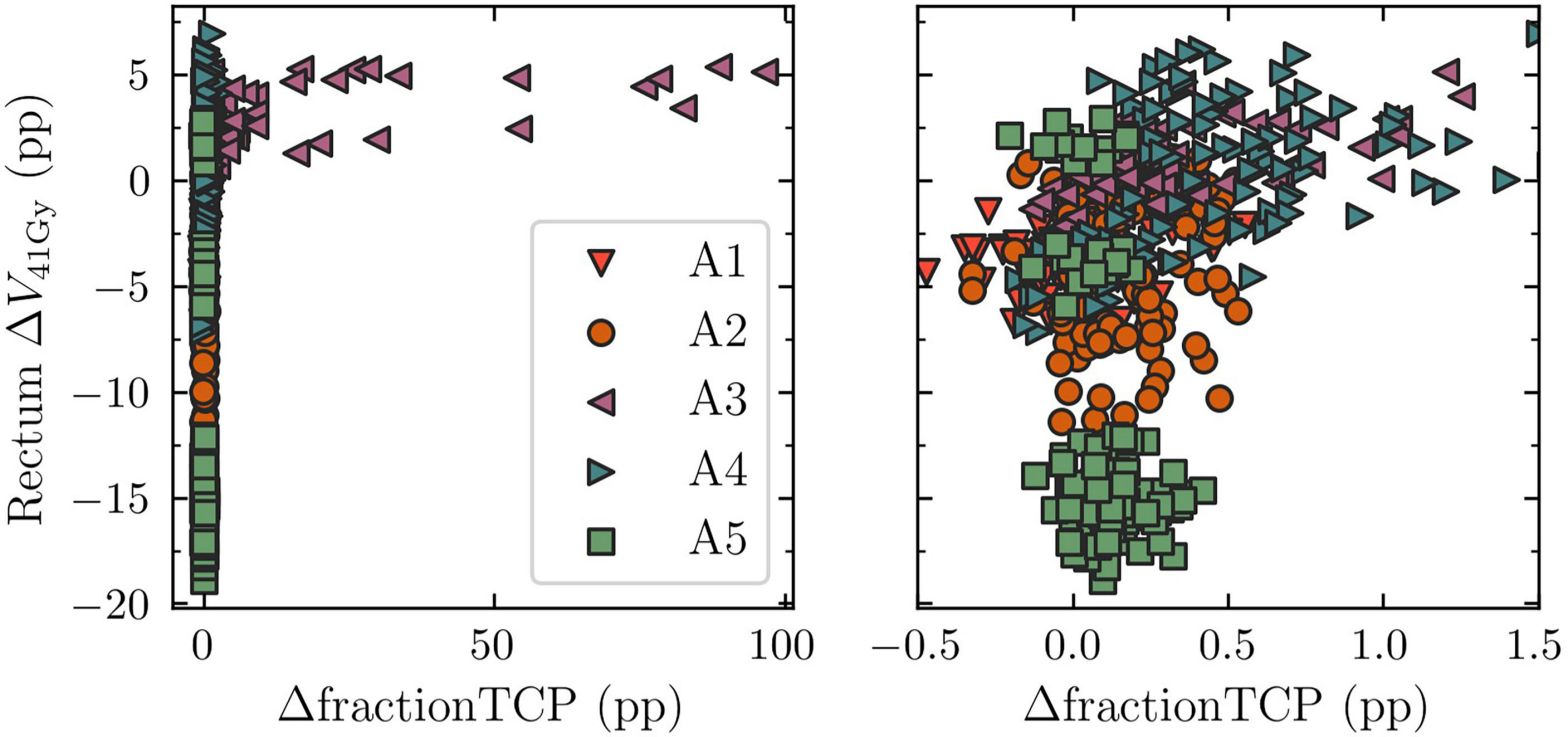
Comparison of rectum *V*_41Gy_ and TCP between DPBN-adap and DPBN-conv. The results have been grouped according to patient anatomy A1-A5. The right and left panel contain the same data but have different horizontal scales. For anatomies A3 and A4 the difference in TCP between the two workflows was relatively large. DPBN-adap resulted in higher rectum dose for A3 and A4, but ‘used’ the extra rectum load to avoid target misses. Δ*V_41Gy=_* (V*_41Gy_*)_adap_-(V*_41Gy_*)_conv,_ ΔfractionTCP=fractionTCP_adap_-fractionTCP_conv,_ pp = percentage points.

The endpoints calculated based on accumulated dose from 7 fractions resulted in several rectum constraint violations even though we imposed hard constraints in the optimization. This was not expected for the DPBN-adap arm; the failure to meet these constraints was attributed to the dose mapping procedure. To eliminate any uncertainty associated with dose mapping, we calculated TCP and rectum DVH endpoints for each treatment fraction. Since the TCP model and DVH metrics are based on the total dose from 7 fractions, we scaled the fraction doses accordingly prior to the endpoint calculations. In **Figure 6**, the difference between DPBN-adap and DPBN-conv in TCP and rectum *V*_41Gy_ is shown for all cases and treatment fractions; the results have been grouped according to anatomy A1-A5 (for each anatomy there are 15x7 = 105 data points). The analysis reveals a marked difference between DPBN-adap and DPBN-conv for some fractions belonging to anatomy A3, for which the difference in fraction specific TCP was larger than 95 percentage points. For most cases and treatment fractions, the difference in TCP was small. It appears that the difference in rectum load between the two workflows depends to a large extent on patient anatomy.

**Figure 7:**
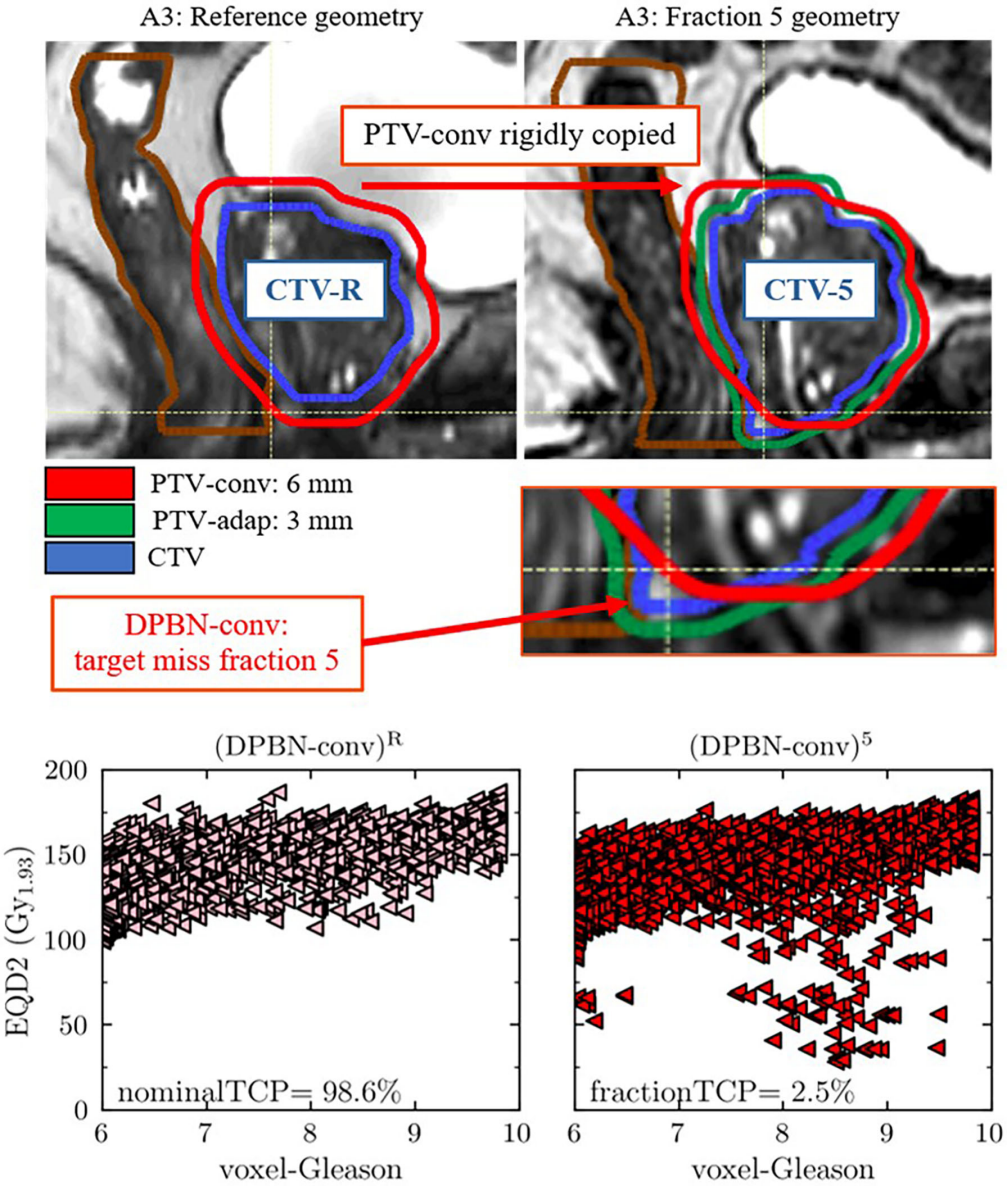
The upper panels show an obvious target miss in the DPBN-conv arm for patient anatomy A3 in fraction 5. The fraction specific CTV-5 is clearly outside of the conventional PTV-conv. The fraction specific TCP, calculated as if the fraction in question was delivered for an entire treatment, was 2.5% compared to 98.6% in the reference plan (DPBN-conv)^R^. The lower panels show the biological dose (EQD2) that was planned (DPBN-conv)^R^ (lower left panel) and delivered in fraction 5 (DPBN-conv)^5^ (lower right panel) to each voxel of the prostate, as a function of predicted Gleason score. The corresponding DPBN-adap plan resulted in 99% TCP.

For cases belonging to A3, for which the adaptive workflow resulted in higher rectum load, the increase in rectum load resulted from adaptive avoidance of target misses to maintain the high TCP of ~99%. A target miss in the conventional workflow is illustrated in **Figure 7**. For the particular case illustrated, the nominal reference TCP was 98.6%, whereas the TCP for fraction 5 was 2.5% (the corresponding DPBN-adap fraction resulted in 99% TCP). The target misses suggest that the conventional margins used were insufficient to account for the interfraction prostate rotations- and deformations of anatomy A3.

### 3.4 Two mechanisms explaining rectum constraint violations in the DPBN arms

**Figure 8:**
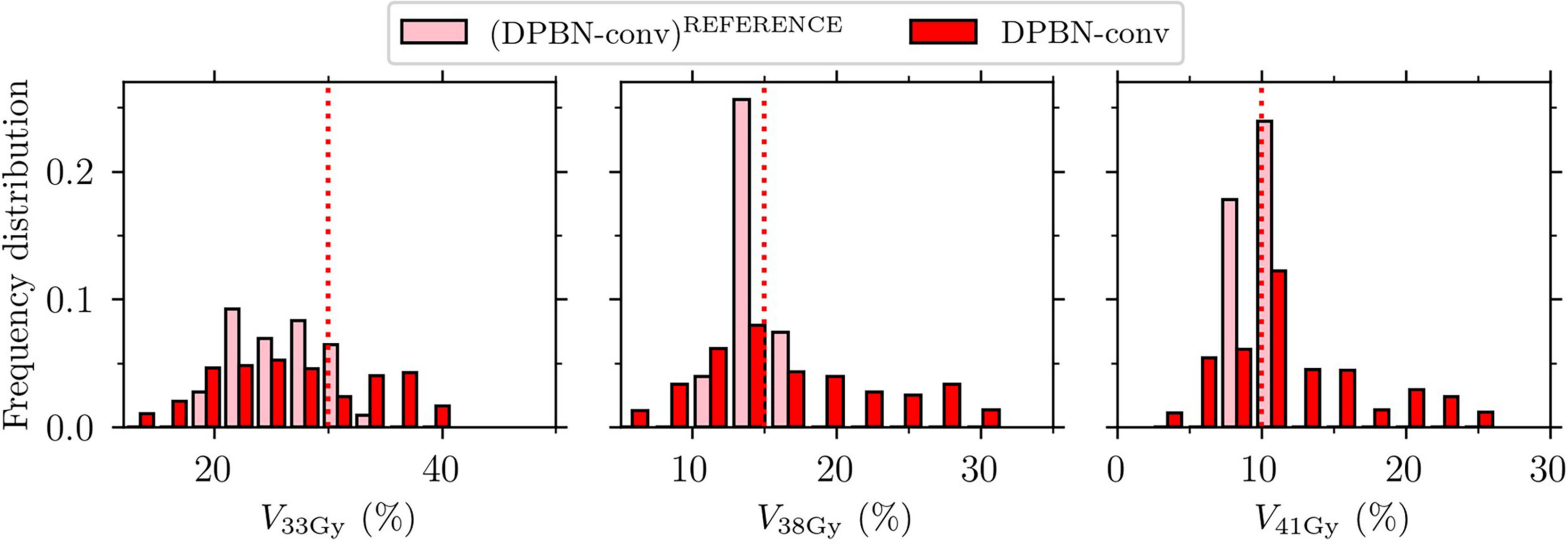
Rectum VaD for the conventional dose painted reference plans (DPBN-conv)^REFERENCE^ and the recalculated fraction doses DPBN-conv. Rectum constrains (indicated by the vertical dotted lines) were violated for the evaluated fraction doses, even though the constraints were met in the nominal reference plans.

**Figure 9:**
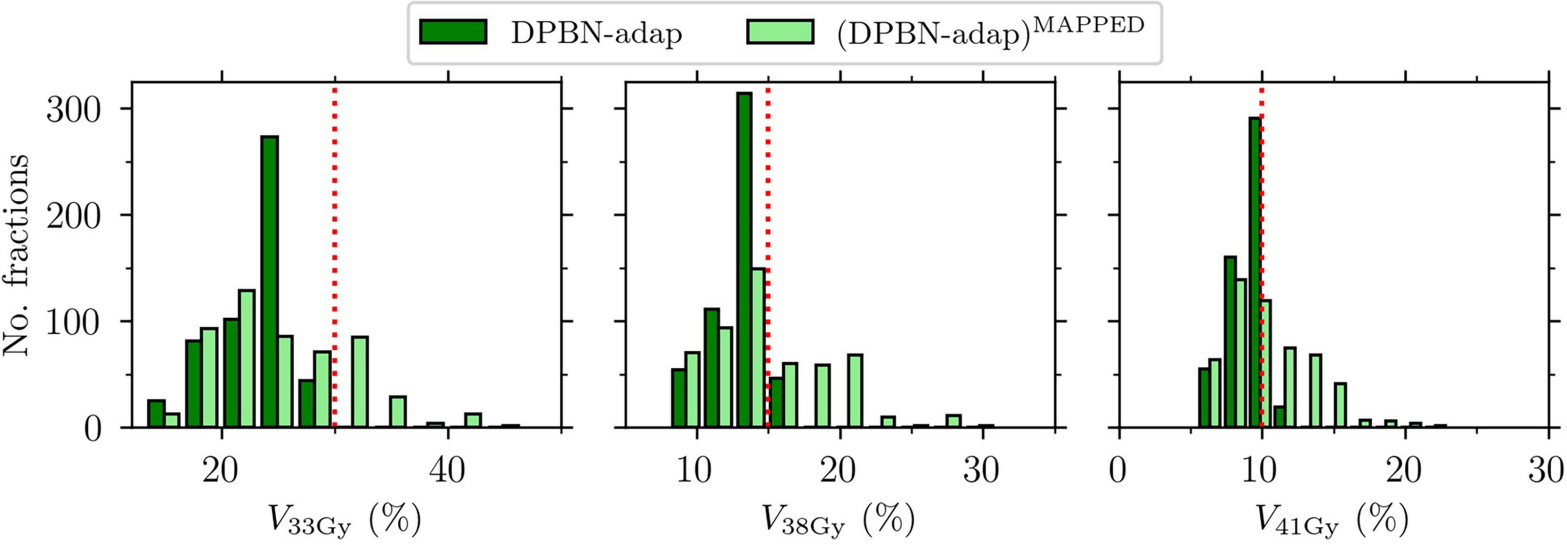
Rectum VaD for the adaptive dose painted fraction doses DPBN-adap and the corresponding mapped fraction doses (DPBN-adap)^MAPPED^. Rectum constrains (indicated by the vertical dotted lines) were violated after dose mapping.

Since hard rectum constraints were violated for several cases despite the intention to treat-to-tolerance, we wanted to explain how this came about, and at the same time verify that no mistakes had entered the simulation pipeline. To this end, we looked at two separate operations in the pipeline: 1) *recalculation of DPBN-conv plans*, and 2) *dose mapping of DPBN-adap plans to the reference geometry*. As our analysis reveals, the optimized treatment plans in fact met the imposed rectum constraints (illustrated in **Figures 8** and **9**, respectively), but the two operations independently altered the *planned* rectum DVH metrics. In **Figure 11**, the relationship between rectal volume changes, and the change in rectum DVH metrics is illustrated. The recalculation operation—which *simulates* the delivery of a conventional reference plan on the anatomy-of-the-day—makes apparent the complexity and limitations in planning and evaluating treatments using dose-volume metrics for organs that experience significant volume changes during the course of therapy; for consistency between planned and delivered dose, the reference geometry must be representative of the whole treatment course.

The dose mapping operation was implemented to be able to accumulate dose at the voxel level, and ultimately to evaluate the total dose from a full (simulated) treatment course. By comparing each fraction dose *before and after* dose mapping, we found that the rectum DVH metrics were not robust to such transformations (**Figure 9**). Accumulation of dose to organs that experience significant volume changes during the course of therapy is a difficult problem that needs attention.

#### 3.4.1 Evaluation of deformable image registrations using Dice score and Hausdorff distance

**Figure 10:**
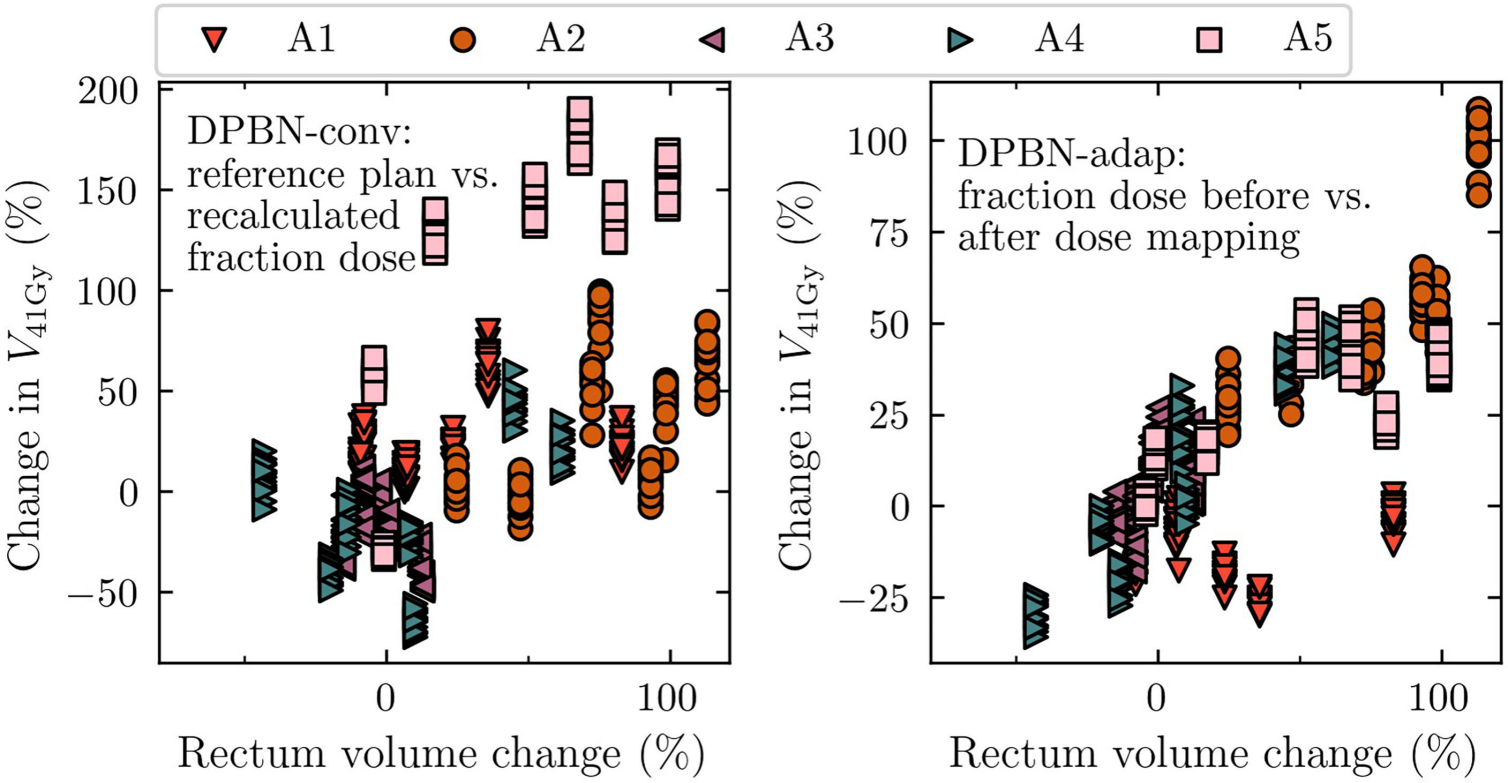
Relative change in rectum *V*_41Gy_ as a function of relative change in rectum volume (compared to the reference rectum volume delineated on the pre-treatment image). The 75 cases have been grouped according to anatomy A1-A5. The left panel shows the effect of recalculating the conventional dose painted reference plans on each fraction geometry, whereas the right panel shows the effect of mapping the adaptive dose painted fraction doses to the reference geometry.

**Figure 11:**
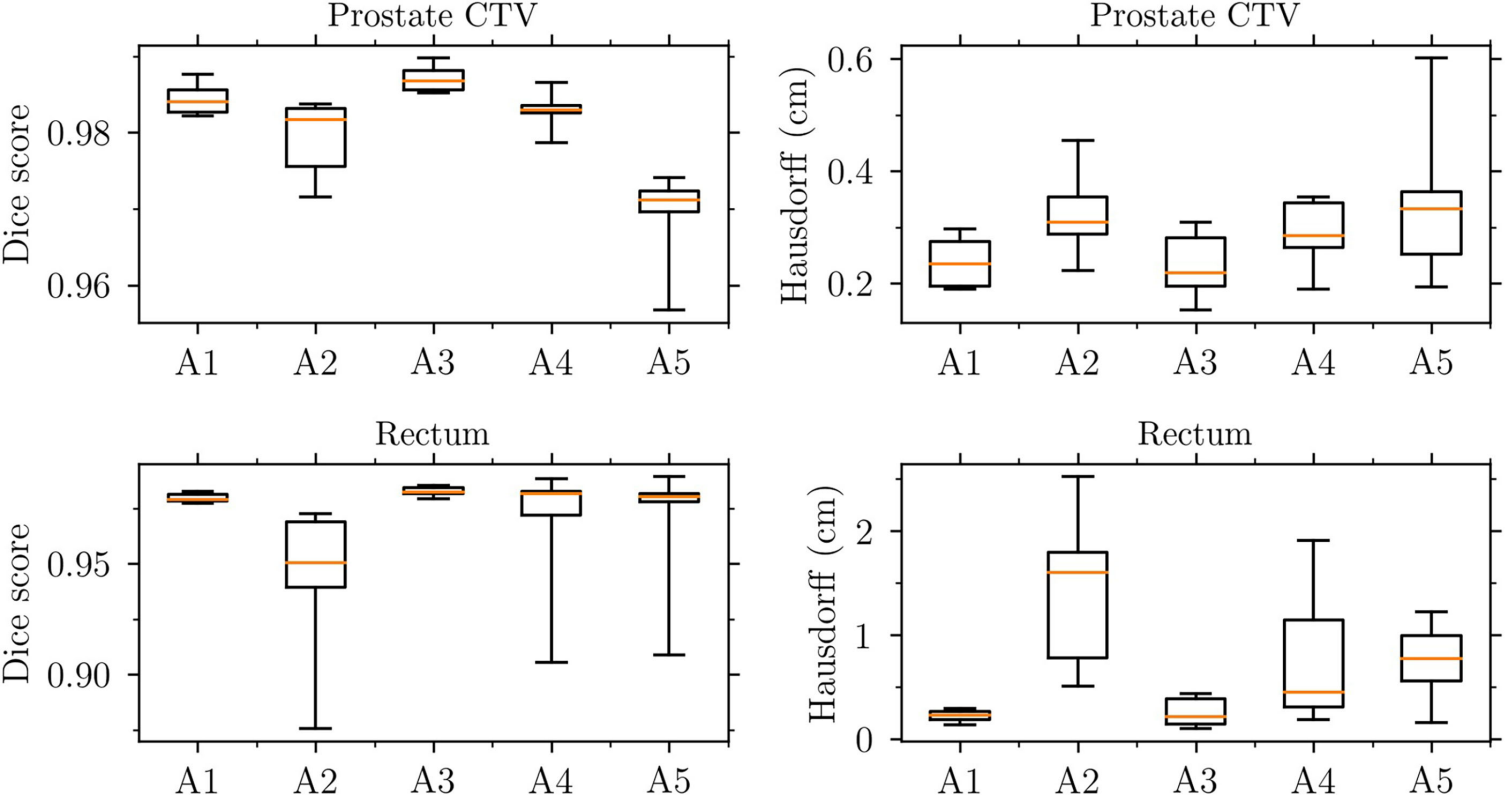
Evaluation of the deformable image registrations used for dose mapping. Dice score (left panels) and Hausdorff distance (right panels) for the prostate CTV (upper panels) and rectum (lower panels).

Dose accumulation using DIR may lead to inaccurate dosimetric evaluation of treatments due to geometric errors in the DIR. Therefore, the DIRs were checked using two common geometrical properties, the DSC and Hausdorff distance. **Figure 10** shows the level of agreement for the prostate CTV and rectum structures for each treatment fraction and patient anatomy. The relatively low DSC values and large Hausdorff distances of the rectum for patient A2 can help explain why the change in *V*_41Gy_ (as illustrated in **Figure 11**) after dose mapping was relatively large for cases belonging to patient A2 (for which the rectum size doubled in one fraction compared to reference). An explanation for the fact that contours do not agree perfectly may be the use of multiple, potentially conflicting, guiding structures, and the fact that we used a combination of guiding structures and image information. It is important to note that, even in the case of *perfect registration of structures* (high DSC and low Hausdorff distance), the change in rectum DVH metrics may still be substantial.

## 4 Discussion

A motivation behind our study were the results from the simulation study by Grönlund et al. (11) showing that there is a relationship between *geometric dose delivery uncertainties* and *the potential TCP gains from dose painting*. Adaptive RT is one possible strategy to reduce those uncertainties. Given the simulations with daily replanning in the present study, only small differences in terms of TCP were observed between adaptive and conventional dose painting. However, a notable difference was observed for some treatment fractions where the target was partially missed in the conventional workflow. Such target misses—especially if they are systematic—could very well compromise tumor control and ultimately the outcome for the patient motivating the use of positional feedback and adaptive measures.

TCP gains from dose escalation (in the studied range of 44 Gy to 60 Gy) were substantially larger compared to the TCP gains observed from dose painting alone; the flatness of the dose-response curve at high target doses yields diminishing returns for the additional cost of redistributing the dose using local radiation sensitivity information. The demonstrated gains are likely clinically insignificant but compared to homogeneous dose escalation, dose painting prescriptions have the potential to decrease dose to the rectum while achieving similar or slightly larger probability for tumor control. Similar advances are likely also for prostate SBRT where narrow margins and steeper dose gradients are adopted to spare the rectum, while peaked dose distributions (higher dose maximum inside the PTV) are likely to increase TCP. However, the positions of dose maxima do not—in general—coincide with radiation resistant foci.

A different approach to lowering rectal doses (and reducing target motion) is to utilize a displacement device (e.g., hydrogel) to physically increase the space between the prostate and rectum (31, 32) but we have not considered such means in our planning study. The rectum DVH metrics used in this work were adapted from a clinical protocol with a homogeneous prescription dose of 42.7 Gy. The validity in using these metrics during dose escalation can be questioned. Moreover, the use of relative rectum VaD, as inherited from the current clinical practice, could potentially contribute to suboptimal results for the adaptive arms; an increased rectum volume a particular day (compared to reference) would allow for an increased absolute rectum volume receiving dose that day. On the contrary, conventional planning is oblivious to future gastrointestinal states. In other words, information about potential interfraction rectum deformations is unknown at the time of conventional planning, potentially resulting in lower-than-planned rectum doses and target misses. In our fraction-by-fraction analysis we indeed observed such results: for cases belonging to two of the patient anatomies, adaptive dose painting resulted in higher rectum doses compared to conventional dose painting; for these cases the extra rectum load was balanced by a relatively high TCP increase due to adaptive avoidance of target misses. Additionally, for some cases, substantial loss of SV target coverage was observed in the conventional workflow, whereas in the adaptive workflow loss of target coverage could be avoided. For patient anatomy A5, the SV target coverage (*V*_95%_) was as low as 53% in one fraction in the homogeneous conventional workflow (prostate prescription dose 44 Gy), whereas the adaptive workflow resulted in an SV target coverage of *V*_95%_=98%. The corresponding control probabilities of the vesicle volume were 85% and 100% for the conventional and adaptive workflow, respectively.

The combination of using ADC maps from one set of patients and applying them to a second set of patients enabled the current study (to our knowledge, this approach has not been used by others). However, the mapping of ADC values from one patient using DIR to a second patient results in a new ADC map due to the different shapes and sizes of the CTVs. The geometric accuracy of the DIRs was checked but the deformed ADC maps used for optimization of DPBN plans were hence not from actual patients. Nevertheless, the resulting ADC maps were visually checked, and the ADC histograms were compared before and after deformation (to ensure that the distributions of ADC values were consistent). We thus conclude that the spatial ADC distributions were realistic enough to investigate the potential of different dose painting strategies. The accuracy and precision with which functional imaging can be used to map out radiation resistant foci will influence the potential of DPBN strategies to increase TCP (11); the model used in this work has inherent limitations related to the uncertainty range of the ADC-to-Gleason mapping function. Further studies, exploring the potential of DPBN, should be conducted in parallel with technological advances in functional imaging and emerging knowledge on potential biomarkers for identifying radiation resistant foci.

The use of relative rectum volumes may confound the relationship between rectal wall doses and induced rectal toxicities. Future studies should be set up to improve the limitations related to the use of relative volumes; perhaps, in the era of daily replanning, one should focus on the matter that matters (e.g., the rectal wall) instead of scoring dose to feces. Future studies should also investigate the potential benefit of dose painting at different target dose levels along the dose-response curve since at low mean doses—where the gradient of the dose-response curve is higher—larger increases in TCP can in theory be achieved with DPBN (7).

## Data availability statement

The raw data supporting the conclusions of this article will be made available by the authors, without undue reservation.

## Ethics statement

The studies involving human participants were reviewed and approved by Etiksprövningsmyndigheten, Uppsala. The patients/participants provided their written informed consent to participate in this study.

## Author contributions

All authors contributed equally to the study design. The corresponding author implemented the simulation pipeline, performed optimizations, and processed the resulting data. All authors contributed equally to the analysis as well as writing the manuscript. All authors contributed to the article and approved the submitted version.

## Acknowledgments

We acknowledge Agnes Angerud and Erik Traneus at RaySearch Laboratories for their help with the implementation of the MRL beam model and TCP objective in RayStation. Tufve Nyholm and Camilla Thellenberg are acknowledged for providing access to part of the image data for the PARAPLY trial.

## Conflict of interest

DT is employed part time by Elekta. However, this work was carried out solely as part of his employment with Uppsala University Hospital and affiliation with Uppsala University.

The remaining authors declare that the research was conducted in the absence of any commercial or financial relationships that could be construed as a potential conflict of interest.

## Publisher’s note

All claims expressed in this article are solely those of the authors and do not necessarily represent those of their affiliated organizations, or those of the publisher, the editors and the reviewers. Any product that may be evaluated in this article, or claim that may be made by its manufacturer, is not guaranteed or endorsed by the publisher.

## Appendix A

### Grönlund's TCP formalism

We applied the failure-driven TCP model for PCa derived by Grönlund et al. (7, 11). They defined TCP in terms of recurrence-free survival at five years using GS as a single variable for dose-response differentiation. Clinical endpoint and GS data from a patient cohort treated with photons (2 Gy x 25) and protons (5 Gy x 4) were used to derive TCP model parameter values. The total dose was converted to EQD2 assuming an alpha/beta ratio of 1.93 Gy. GS is related to the risk of recurrence (33) and is determined through histological assessment of prostate biopsies sampled prior to treatment as per routine clinical practice. GS is used as a prognostic tool together with the TNM system and prostate-specific antigen (PSA) concentration in blood to classify the cancer into low-, intermediate, or high-risk PCa. For a full derivation of the model, see Grönlund’s previous work (7). In brief, the total control probability is given by a product of voxel control probabilities according to

,
$$\text{TCP}=\prod_{j}{\text{VCP}}_{j},j\;\epsilon \text{ CTV}$$

i. e. it is assumed that potential bystander effects can be neglected. To establish a direct link between image intensities and TCP they used published correlations between ADC and GS to construct a mapping from ADC to Gleason probabilities according to

$$p(G_{k}|ADC)=\frac{p\left(ADC|G_{k}\right)}{\sum_{m}p\left(ADC|G_{m}\right)}$$

here *_p_(ADC|G_k_)* are lognormal distributions of ADC values for each Gleason score category.*G_k_* The 25th and 75th percentiles of the lognormal distributions were set to correspond to the uncertainty ranges found in the work by Turkbey et al. (8). The voxel control probability *TCP_j_* of the j:th voxel is given by a geometric average

$${\text{VCP}}_{j}\left(d_{j},\text{ADC}\right)={\left(\prod_{k}R{\left(d_{j,}G_{k}\right)}^{p(G_{k}\text{|ADC})}\right)}^{v_{k}}$$

ver a set of dose-response functions *R(d_j,_ G_k_)*defined as

$$R\left(d_{j,}G_{k}\right)=1/{\left(1+\frac{D_{50}\left(G_{k}\right)\;}{d_{j}}\right)}^{4\gamma_{50}}$$

,here *d_j_* is the dose delivered to voxel *j*, v*_j_* is the fractional volume of the voxel within the prostate contour, *D_50_(G_k_)* is the dose level corresponding to 50% control probability, and γ_50_ is the normalized dose-response gradient (assumed to be independent of GS) evaluated at *D_50_*. These parameter values were derived based on the assumption of a linear relationship between GS and voxel control probability at the homogeneous dose level prescribed to the patient cohort under study (EQD2 = 91.6 Gy_1.93_). Normal tissue voxels were treated as Gleason score 6.

In contrast to previous studies by Grönlund et al. (7, 11) we included the seminal vesicles in the TCP model to be able to include the effects of potential loss of target coverage. In previous studies, the seminal vesicles were simply assumed to be controlled by the dose level they received (i.e. TCP_ves_=100%). In the present work, we treated the full SV volume as GS 6 by assigning to each SV voxel the highest possible ADC value in the ADC-to-Gleason mapping range.
